# High probability of comorbidities in bronchial asthma in Germany

**DOI:** 10.1038/s41533-017-0026-x

**Published:** 2017-04-21

**Authors:** S. Heck, S. Al-Shobash, D. Rapp, D. D. Le, A. Omlor, A. Bekhit, M. Flaig, B. Al-Kadah, W. Herian, R. Bals, S. Wagenpfeil, Q. T. Dinh

**Affiliations:** 1 Department of Experimental Pneumology and Allergology, Saarland University Faculty of Medicine, Homburg/Saar, Germany; 2 Department of Biometry, Epidemiology and Clinical informatics, Saarland University Faculty of Medicine, Homburg/Saar, Germany; 3 Department of Internal Medicine V, Pneumology, Allergology and Respiratory Critical Care Medicine, Saarland University Faculty of Medicine, Homburg/Saar, Germany; 4 Department of Otorhinolaryngology, Saarland University Faculty of Medicine, Homburg/Saar, Germany; 5 Head of the Regulatory Management Division, Association of Statutory Health Insurance Physicians Saarland, Saarbrucken, Germany

## Abstract

Clinical experience has shown that allergic and non-allergic respiratory, metabolic, mental, and cardiovascular disorders sometimes coexist with bronchial asthma. However, no study has been carried out that calculates the chance of manifestation of these disorders with bronchial asthma in Saarland and Rhineland-Palatinate, Germany. Using ICD10 diagnoses from health care institutions, the present study systematically analyzed the co-prevalence and odds ratios of comorbidities in the asthma population in Germany. The odds ratios were adjusted for age and sex for all comorbidities for patients with asthma vs. without asthma. Bronchial asthma was strongly associated with allergic and with a lesser extent to non-allergic comorbidities: OR 7.02 (95%CI:6.83–7.22) for allergic rhinitis; OR 4.98 (95%CI:4.67–5.32) allergic conjunctivitis; OR 2.41 (95%CI:2.33–2.52) atopic dermatitis; OR 2.47 (95%CI:2.16–2.82) food allergy, and OR 1.69 (95%CI:1.61–1.78) drug allergy. Interestingly, increased ORs were found for respiratory diseases: 2.06 (95%CI:1.64–2.58) vocal dysfunction; 1.83 (95%CI:1.74–1.92) pneumonia; 1.78 (95%CI:1.73–1.84) sinusitis; 1.71 (95%CI:1.65–1.78) rhinopharyngitis; 2.55 (95%CI:2.03–3.19) obstructive sleep apnea; 1.42 (95%CI:1.25–1.61) pulmonary embolism, and 3.75 (95%CI:1.64–8.53) bronchopulmonary aspergillosis. Asthmatics also suffer from psychiatric, metabolic, cardiac or other comorbidities. Myocardial infarction (OR 0.86, 95%CI:0.79–0.94) did not coexist with asthma. Based on the calculated chances of manifestation for these comorbidities, especially allergic and respiratory, to a lesser extent also metabolic, cardiovascular, and mental disorders should be taken into consideration in the diagnostic and treatment strategy of bronchial asthma.

## Bronchial asthma: Prevalence of co-existing diseases in Germany

Patients in Germany with bronchial asthma are highly likely to suffer from co-existing diseases and their treatments should reflect this. Quoc Thai Dinh at Saarland University Hospital in Homburg, Germany, and co-workers conducted a large-scale study of patients presenting with bronchial asthma in the Saarland region between 2009 and 2012. Patients with asthma made up 5.4% of the region’s total population, with a higher prevalence occurring in females. They found that bronchial asthma was strongly associated with allergic comorbidities such as rhinitis. Indeed, asthmatic patients had a seven times higher chance to suffer from allergic rhinitis than the rest of the population, and were at higher risk of respiratory diseases like pneumonia and obstructive sleep apnea syndrome. Further associations included cardiovascular, metabolic and mental disorders. Dinh’s team call for asthma treatments to take such comorbidities into account.

## Introduction

Bronchial asthma has become widespread and currently affects almost a quarter billion people worldwide with an increasing rate of prevalence. It is a heterogenic, complex, chronic inflammatory and obstructive lung disease, which can be associated with many comorbidities.^1–6^ While clinical experience suggests an association between asthma and comorbidities such as rhinitis, gastro-esophageal reflux disease (GERD), and psychiatric comorbidities, this link is still controversial. In contrast to chronic obstructive pulmonary disease (COPD), the association between asthma and comorbidities in two German regions (Saarland and Rhineland-Palatinate) has not been investigated intensively so far.

Traditionally, bronchial asthma has been divided into several subtypes that are characterized as extrinsic (allergic) or intrinsic (non-allergic) asthma and bronchial asthma with a mixed form of intrinsic and extrinsic asthma.^7, 8^ Today, bronchial asthma can be classified into many either T_H_2-dependent or non-T_H_2-dependent phenotypes.^8^ For diagnosis, the ICD-10 code is applicable to four different subtypes for bronchial asthma: extrinsic asthma (J45.1), intrinsic asthma (J45.0), a mixed form of intrinsic and extrinsic asthma (J45.8), and “not-otherwise specified” asthma (J45.9).^9^

## Results

### Asthma prevalence rates and comorbidities

Medically insured people living in Saarland and suffering from any form of asthma represented 5.4% of the overall population in 2011. The prevalence of asthma in males in 2011 was 5.1% and 6.0% in females.

The distribution of asthma between the two genders varied according to the affiliation to an age group. In patients who were under 18 years of age, the prevalence of asthma was higher in males (7.64%) compared to females (5.12%). This relation shifted in patients aged between 18 and 60 (males: 3.39%, females: 5.26%) and ones, aged 61 and older (males: 3.62%, females: 4.78%).

### Allergic diseases

**Fig. 1:**
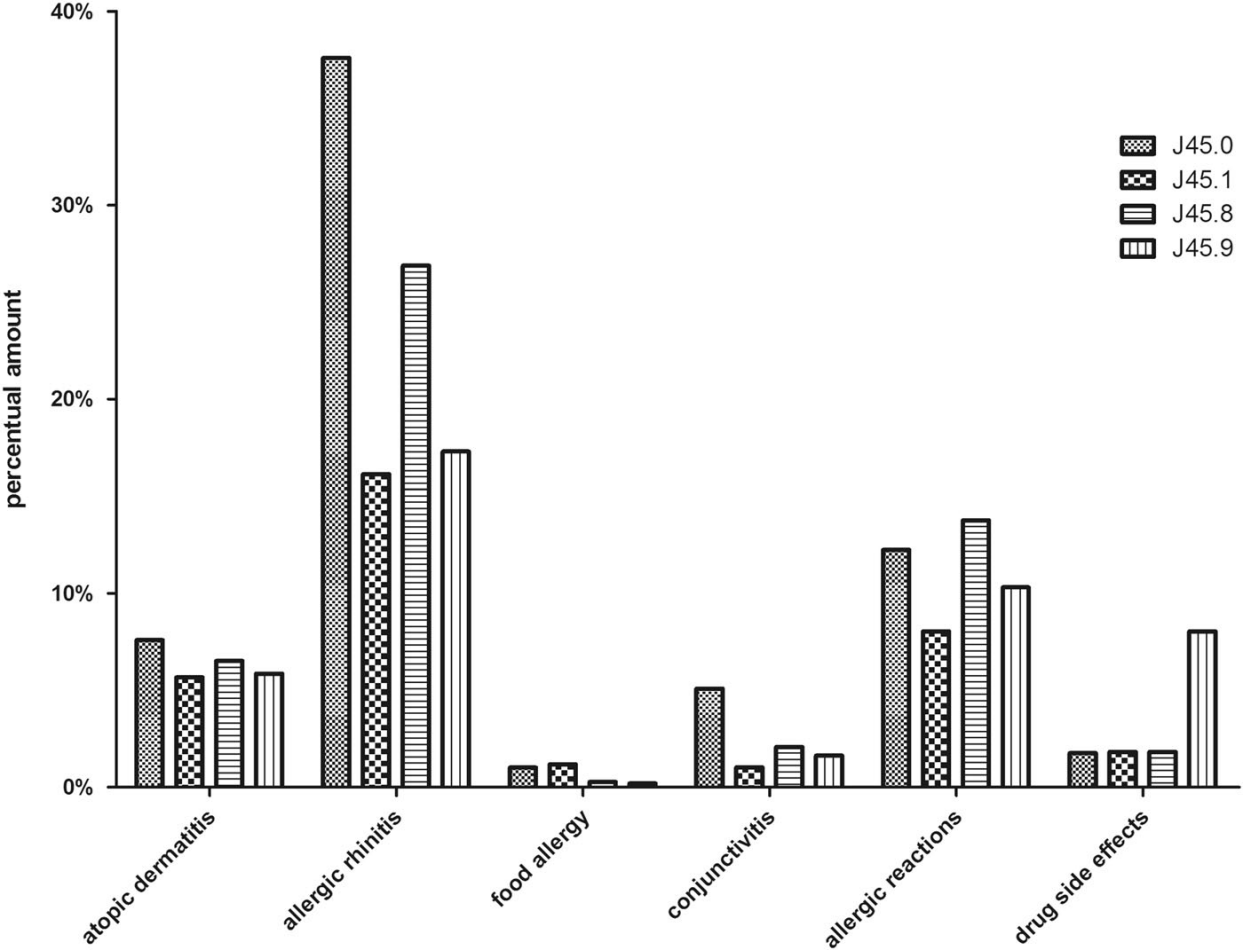
Bronchial asthma subtypes and allergic comorbidities: Percentage of patients suffering from an asthma subtype and at least one allergic comorbidity in Saarland in 2011

One in four asthma patients in Saarland also suffered from allergic rhinitis (24.18%); about one in 10 suffered from unspecified allergic reactions (11.05%) (Fig. 1). Atopic dermatitis affected 6.43%, allergic conjunctivitis 2.65%, drug allergy 1.66%, and food allergy 0.53%, which was less than 3% of all asthma patients in Saarland. Allergic bronchopulmonary aspergillosis was almost non-existent in the population of Saarland’s asthmatics (lower than 0.01% in all four asthma subtypes).

Allergic rhinitis was the comorbidity that appeared most often in combination with allergic asthma (Fig. 1). The percent amount was 37.61%. It appeared less often in combination with the three other asthma diagnoses (allergic rhinitis in J45.1: 16.13; J45.8: 26.89; J45.9: 17.3%). The second most common comorbidity was an allergic reaction, which was not otherwise specified. A substantial proportion of asthmatics (13.78%) with the mixed phenotype also suffered from unspecified allergies, the number of patients with other asthma types was lower (J45.0: 12.25%; J45.1: 8.04%; J45.9: 10.32%). Atopic dermatitis was slightly increased in patients with allergic asthma (7.6%) compared to patients with non-allergic asthma (5.67%), the mixed form (6.52%), and not otherwise specified asthma (5.87%). The distribution of the allergic comorbidities varied between the two genders. Women were more often affected than men.

### Diseases of the respiratory system

**Fig. 2:**
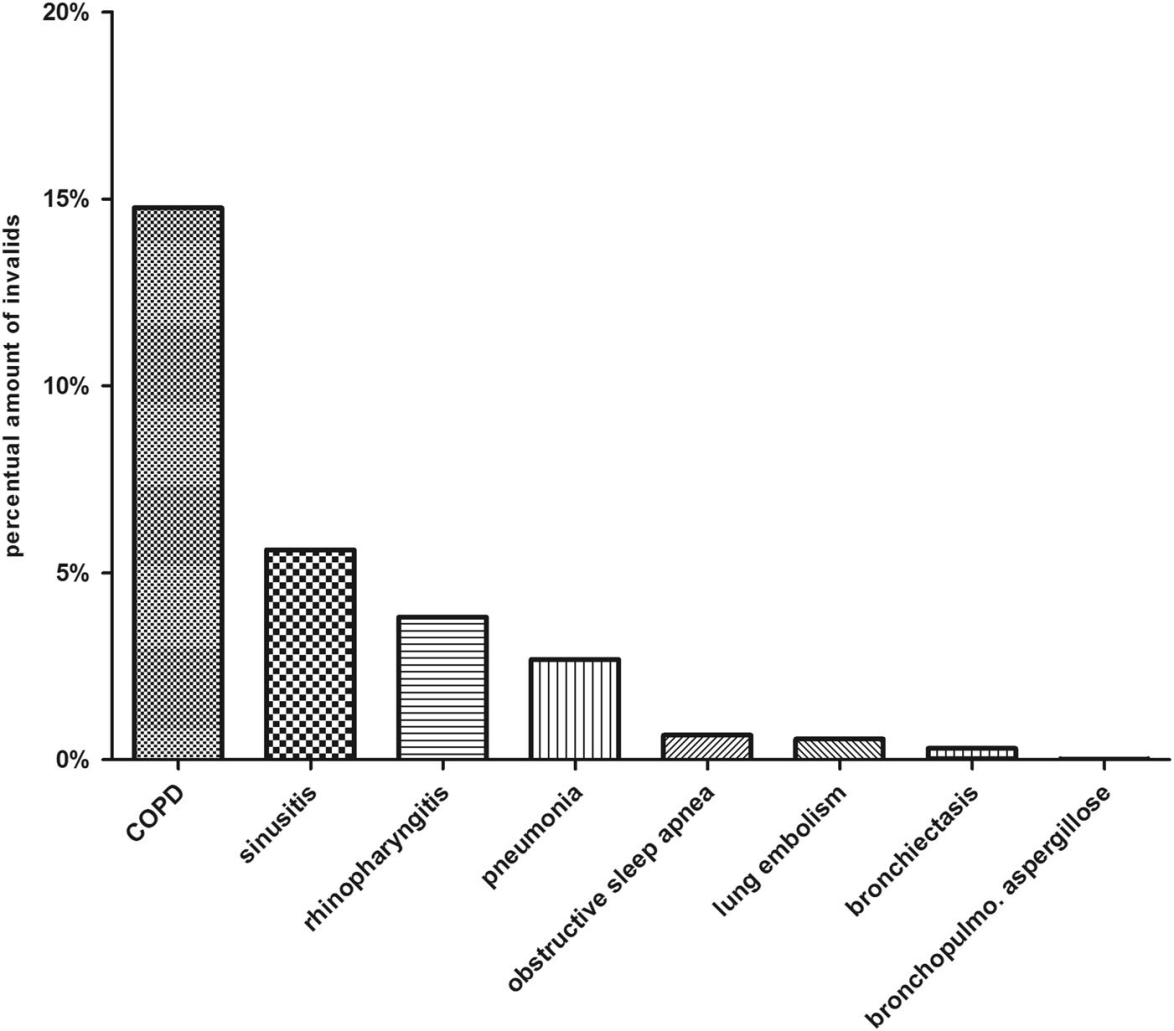
Respiratory diseases in the context of asthma: Respiratory comorbidities in combination with bronchial asthma in Saarland in 2011

Our study showed that about 15% of all asthmatics in Saarland also suffered from COPD. The other comorbidities concerning the respiratory system occurred more rarely; only the percentage of acute and chronic sinusitis was higher than 5% (Fig. 2).

### Cardiovascular diseases

**Fig. 3:**
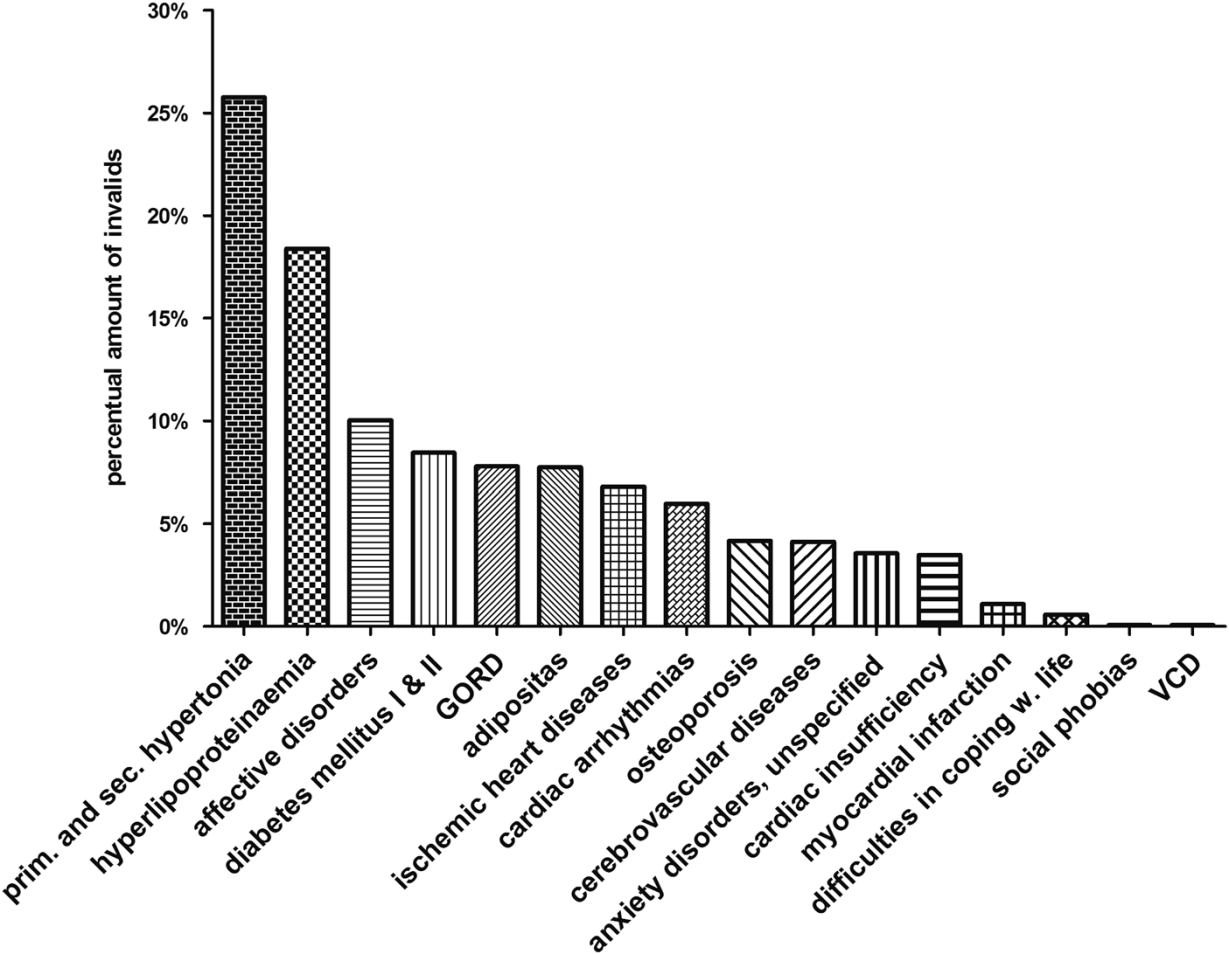
Asthma and cardiovascular, metabolic, cerebrovascular and psychosomatic diseases: Percentage of patients suffering from *c*ardiovascular, metabolic, cerebrovascular or psychosomatic diseases in combination with bronchial asthma in 2011

About 25% of all patients suffering from bronchial asthma also had to deal with primary and secondary hypertonia (Fig. 3). Altogether, hypertonia was the cardiovascular comorbidity most often emerging in the context of bronchial asthma. Ischemic heart diseases and cardiac insufficiency were lower in allergic asthma compared to the other asthma forms; cardiac arrhythmias, and myocardial infarction was diagnosed consistently among the different asthma phenotypes (Fig. 3).

### Metabolic diseases

Metabolic diseases also occurred in combination with bronchial asthma (Fig. 3). The most common one was hyperlipoproteinaemia, which appeared in 18.38%. Both types of diabetes mellitus—type I and type II—emerged in 8.44% and adiposity affected 7.72% of all asthmatics in Saarland.

### Mental disorders

Patients with bronchial asthma in some cases also suffered from mental illnesses (Fig. 3). The most frequent mental illnesses were affective disorders (10.02%) followed by unspecified anxiety disorders (3.57%). Difficulties in coping with life (0.57%) and especially social phobias (0.065%) were virtually nonexistent.

### Other diseases

Cerebrovascular diseases, which described subarachnoid, intracerebral and other, non-traumatic, intracranial hemorrhages and strokes, added up to 4.12% of all asthmatics in the defined timeframe. GERD affected 7.78% of all asthmatics and was therewith much more frequent in asthma patients than osteoporosis at 4.16% and vocal cord dysfunction (VCD), which was 0.06% (Fig. 3).

### Odds ratios for asthma and comorbidities

#### Allergic diseases

**Fig. 4:**
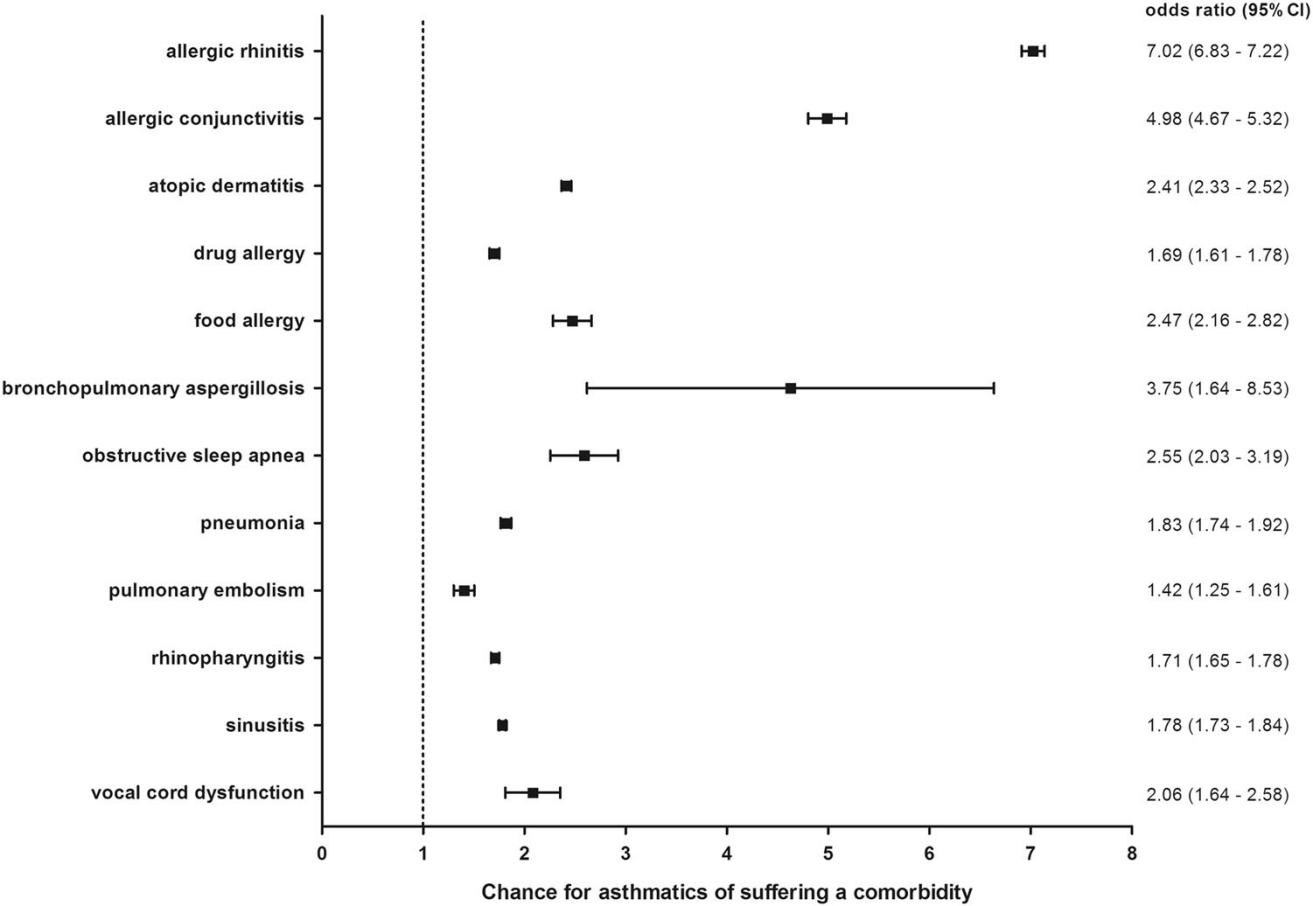
Allergies and respiratory diseases: Sex- and age-adjusted odds ratios for allergies, respiratory diseases and VCD in the context of bronchial asthma in Saarland and Rhineland-Palatinate in 2012. Bars show confidence intervals

To verify whether the coexistence of the diagnosis found in the present study also had an association with bronchial asthma, an association study was carried out to investigate the odds ratio of the comorbidities in bronchial asthma. For asthmatics, the chance of suffering from one of the analyzed comorbidities varied for each disease. Allergic diseases such as atopic dermatitis or allergic conjunctivitis manifested significantly more often in asthmatics compared to our control group (Fig. 4). The highest odds ratio in the context of bronchial asthma appears in allergic rhinitis with 7.02 (95% CI:6.83–7.22). The chance of suffering from allergic conjunctivitis was 4.98 (95% CI:4.67–5.32) times in asthmatics. The odds ratios of atopic dermatitis (OR 2.41 (95% CI:2.33–2.52)) and food allergy 2.47 (95% CI:2.16–2.82) were at a similar level in asthmatics in Saarland and Rhineland-Palatinate; drug allergy amounted to 1.69 (95% CI:1.61–1.78).

A subgroup analysis of the distinct asthma subtypes revealed slightly different results concerning the chances for the occurrence of a disorder in 2012 (Table 1).

**Table 1:** Odds ratios with 95% confidence intervals for each of the four ICD-10 asthma subtypes: The odds ratios are adjusted for age and sex

	J45.0	J45.1	J45.8	J45.9
Drug allergy	1.84(1.71–1.97)	2.05(1.83–2.29)	1.94(1.74–2.18)	1.6(1.52–1.69)
Allergic conjuunctivitis	7.4(6.94–7.88)	1.74(1.52–2.0)	3.2(2.85–3.59)	2.31(2.17–2.47)
Food allergy	3.89(3.36–4.5)	1.66(1.24–2.24)	2.2(1.66-2.91)	1.6(1.39–1.85)
Atopic dermatitis	2.24(2.14–2.35)	1.66(1.53–1.8)	1.96(1.812.13)	2.08(2.01–2.16)
Allergic rhinitis	9.77(9.48–10.07)	1.54(1.45–1.64)	4.1(3.89–4.32)	3.13(3.05–3.22)

Respectively, the chance of coexistence of extrinsic (allergic) asthma and allergic rhinitis was very high at 9.77 (95% CI:9.48–10.07) and was in contrast to intrinsic (non-allergic) asthma (OR 1.54, 95% CI:1.45–1.64), which represented the lowest chance of coexistence. Asthmatics of the four asthma subtypes also suffered with quite similar odds ratios between 1.66 (95% CI:1.53–1.8) in intrinsic and 2.24 (95% CI:2.14–2.35) in the extrinsic form of asthma from atopic dermatitis. The range of odds ratios for food allergy in the context of bronchial asthma started from 1.6 in unspecified asthma to 3.89 (95% CI:3.36–4.5) in the allergic. Similarly with high odds for allergic rhinitis, the chance of suffering from allergic conjunctivitis was 7.4 times in allergic asthmatics. This chance was much lower in the other subtypes (J45.1: 1.74, 95% CI:1.52–2.0; J45.8: 3.2, 95% CI:2.85–3.59; J45.9: 2.31, 95% CI:2.17–2.47). Although the lower chance of occurrence for a drug allergy in asthmatics is given, there were no obvious differences among the asthma subtypes, which ranged from 1.6 (95% CI:1.52–1.69) in unspecified to 2.05 (95% CI:1.83–2.29) in intrinsic asthma.

#### Respiratory diseases

The odds ratios for the respiratory diseases, which were included in this study, varied between 3.75 (95% CI: 1.64–8.53) for bronchopulmonary aspergillosis and 1.42 (95% CI:1.25–1.61) for pulmonary embolism (Fig. 4). All other respiratory diseases emerged significantly more often in the presence of bronchial asthma.

#### Cardiovascular diseases

**Fig. 5:**
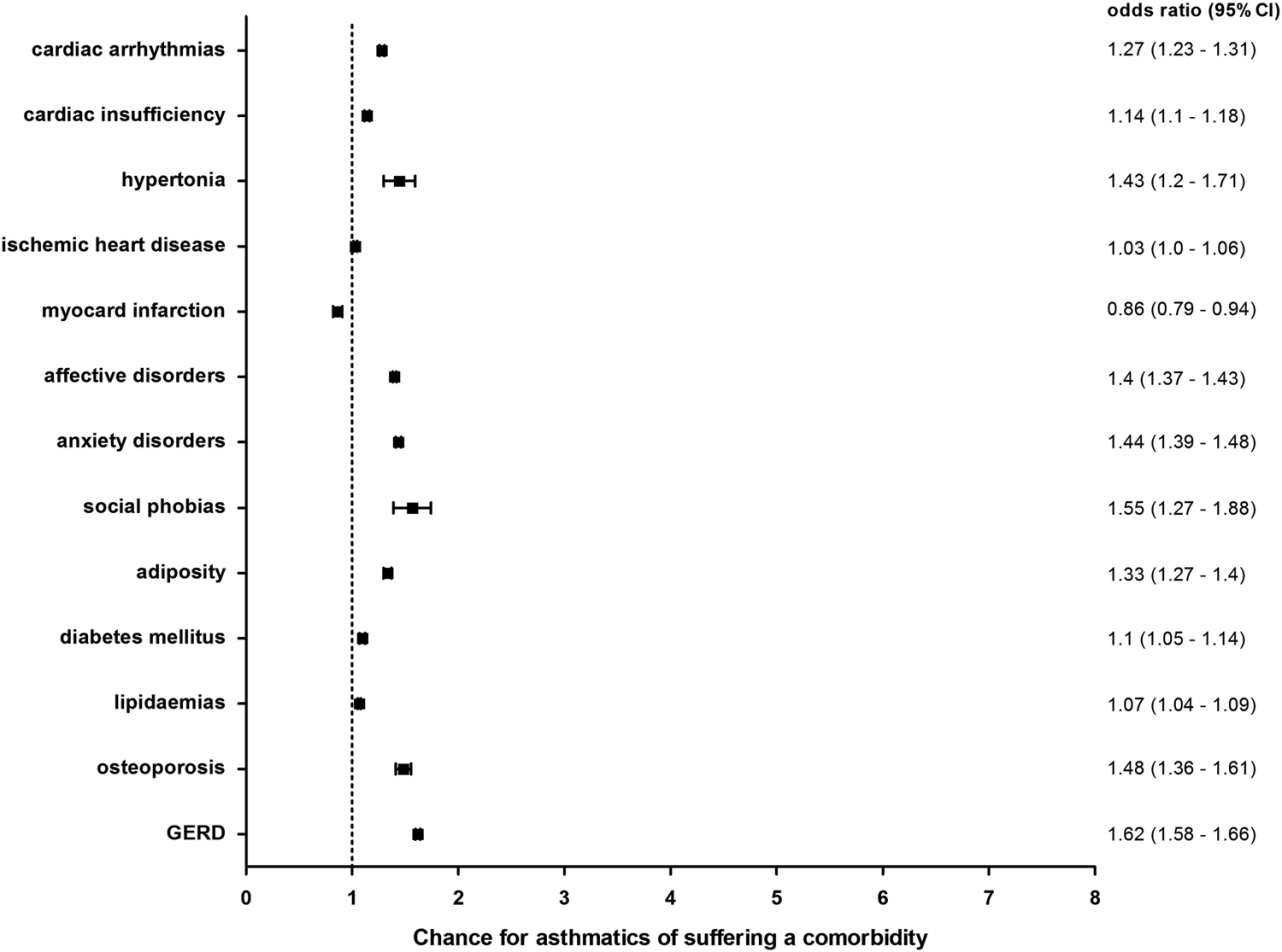
Cardiovascular, mental, metabolic and other comorbidities: Sex- and age-adjusted odds ratios (95% CI) for cardiovascular, mental, metabolic and other diseases in the context of bronchial asthma in Saarland and Rhineland-Palatinate in 2012. Bars show confidence intervals

The chance of suffering from a cardiovascular disease in combination with bronchial asthma varied slightly among the analyzed disorders (Fig. 5). The chance of contracting a primary or secondary arterial hypertonia (OR 1.43, 95% CI:1.2–1.71), a cardiac insufficiency (OR 1.14, 95% CI:1.1–1.18), cardiac arrhythmias (OR 1.27, 95% CI:1.23–1.31) or ischemias (OR 1.03, 95% CI:1.0–1.06) was faintly increased. A myocardial infarction (OR 0.86, 95% CI:0.79–0.94) occurred more rarely in asthmatics compared to controls.

#### Mental disorders

The mental disorders investigated in this study were anxiety disorders, social phobias and affective disorders (Fig. 5). The odds ratios for these mental diseases ranged between 1.4 (95% CI:1.37–1.43) for affective disorders, 1.44 (95% CI:1.39–1.48) for anxiety disorders and 1.55 (95% CI:1.27–1.88) for social phobias.

#### Metabolic diseases

The chance of developing metabolic comorbidities in asthmatics in Saarland and Rhineland-Palatinate in 2012 varied slightly. Adiposity (OR 1.33, 95% CI:1.27–1.4) represented the highest odds ratio. The odds ratios for lipidaemias and diabetes mellitus were a little bit lower (Fig. 5).

#### Other Diseases

The odds ratio for VCD in asthma patients was 2.06 (95% CI:1.64–2.58) (Fig. 4). For osteoporosis 1.48 (95% CI:1.36–1.61) and for GERD (OR 1.62, 95% CI:1.58–1.66) (Fig. 5) the odds ratios were lower.

## Discussion

The prevalence of bronchial asthma in Saarland in 2011 was 5.4%. Our result was slightly higher compared to a study in Bavaria with almost half a million asthma patients, which described a prevalence of 4.8% in females and 4.5% in males^10^ compared to 6.0% in females and 5.1% in males in this study.

Women were more prone to asthma and allergies than men—which covered clinical observations.^11^ There is evidence that genetic factors may trigger asthma and allergies in women.^12^ According to a nationwide telephone survey from 2009, the mainly rural population of the federal state of Saarland did suffer about equally often from asthma compared to the pan-German average.^13^ Other studies determined the prevalence of bronchial asthma as being between 1.8 and 6.34%.^12, 14–16^ In contrast to the pan-German average, the prevalence of asthma in the United Kingdom, New Zealand, and Australia was higher.^17, 18^

The prevalence among age groups was higher for under 18-year-old boys than in age-matched girls, but this imbalance shifted for older patients, and resulted in a higher rate of affliction in women.^19^ There is evidence that puberty may play an important role for the manifestation and further development of asthma. After puberty, boys seem to “grow-out” of asthma, but the reasoning remains unclear. A possible contribution of sexual hormones has not been demonstrated yet.^20^ Moreover not only does the prevalence of asthma change within a gender dependent on the age, but the clinical outcome can also vary.^21^

The optimal therapy for bronchial asthma consists not only of the exhaustion of the existing pharmacological therapies but also the identification and treatment of comorbidities. The present study aimed to analyze the co-existence and association of many different diseases as comorbidities of bronchial asthma.

The large coexistence of diseases, such as hyperlipoproteinaemia and diabetes, with bronchial asthma suggest an association between them, but the analysis of the odds ratio revealed no increased chance, as has already been shown in other studies.^22, 23^ However, asthmatics do have an increased chance of suffering from osteoporosis, pneumonia, vocal cord dysfunction, and obstructive sleep apnea. A recently published study^24^ reported an increased chance of developing obstructive sleep apnea if the patient is asthmatic. Our study results cover these findings. The chance of sleep apnea is 2.55 times in asthmatics compared to non-asthmatics.

Concerning the asthma-related chance of cardiovascular diseases, asthma — as a chronic inflammatory disease—can induce atherosclerosis^24^ by systemic inflammation but does not increase the risk of heart disease in middle-aged adults.^25^ The IgE is itself potentially pro-atherogenic through actions on mast cells and platelets.^26, 27^ In contrast to that result, this association study shows an only slightly increased chance for cardiac ischemia and only a very weak association with cardiac insufficiency or arrhythmia in asthmatics to the same degree as shown in the literature.^22, 24, 28^ The odds ratio of hypertonia in the present study is at the same level as the comparative study in Italy.^22^ The cause of diverse results among different studies may be based on different lifestyles in the analyzed populations. Risk factors such as unhealthy nutrition and consequent adiposity and hypertonia should be taken into consideration when evaluating a population study.

With respect to the high chance of coexistence of asthma with allergic rhinitis, the present study revealed a very high odds ratio of 9.77 (95% CI: 9.48–10.07) for allergic asthma in contrast to 1.54 (95% CI: 1.45–1.64) for intrinsic asthma. Supporting our results, patients with allergic or non-allergic rhinitis were found to have a high chance for also suffering from asthma and conversely chronic rhinosinusitis,^29^ and nasal polyps were often found together in asthmatics. These findings may support the hypothesis of an allergic march.^30–32^ Since the odds ratio of allergic rhinitis was visibly higher in the present study than in the one from Italy^22^ while the chance of coexistence of asthma and rhinosinusitis is similar.

Supporting our findings, food allergy is often observed together with asthma; the underlying mechanism of this comorbidity is diverse.^33–36^ Similar to our results, atopic dermatitis and drug allergy showed a strong association with asthma. Patients with severe atopic dermatitis had a higher chance for the development of asthma. There is an overexpression of Th2 cytokines in lymphatic organs with cutaneous inflammation. Asthma is closely linked to atopic dermatitis; the IgE level is increased in 80% of all patients with atopic dermatitis.^37^

The metabolic syndrome may play a role for the partially increased occurrence of comorbidities like adiposity (see Fig. 5) in asthmatics in Saarland. It has already been shown, that there is a link between adiposity and the risk of asthma^38, 39^ and that the synergy of adiposity and insulin resistance may lead to an increased risk of asthma.^40^ Osteoporosis can be a result of the long-term treatment of asthma with corticosteroids.

It has already been shown that gastro-esophageal reflux disease plays a role in asthma and infectious processes in the respiratory system.^41, 42^ The chance for asthmatics of also suffering from GERD was increased. This seems reasonable as esophagus, trachea, and lungs are adjacent organs with a similar embryologic origin in the nervous system. Epidemiologic studies showed a variable prevalence for GERD in asthmatics between 12 and 85%.^42–46^

The present study demonstrated a slightly increased chance for asthmatics to suffer from anxiety (OR 1.44), social phobias (OR 1.55) and affective disorders (OR 1.40). Clinical experience showed that emotional stress, caused for example by the fear of suffering an asthma attack and being unable to breath properly, can exacerbate asthma and often lead to psychiatric comorbidities.^22, 46^ However, the prevalence of psychological and psychiatric disorders in asthmatics was very diverse between countries because there were no general definitions, and there are differences in the nomenclature and in the small samples of study patients. Mental illnesses were reported to affect asthmatics to a special degree.^47, 48^ Affective disorders also played an important role in the context of asthma.^49, 50^

This study was based on a large quantity of data, which represented its strength. The large quantity of data makes the results more reliable and accurate. There are also some weaknesses which should be mentioned These could be inaccuracies in making a diagnosis by the attending physicians. Accuracy of the ICD-10 diagnosis by practicing physicians may be limited due to prescription and financial purposes so that a question still remains regarding the real validity of diagnosis in health care institutions. Furthermore, the ICD-10 coding system itself encompasses some weaknesses concerning the accuracy and definition of distinct diseases and therefore it needs continuous updates, edits and alignments with the latest state of the art.

## Conclusion

Clinical experience has shown that distinct disorders, such as allergic diseases and respiratory comorbidities, often coexist with bronchial asthma. However, there has not yet been any large-scale study that calculated exactly the probability of comorbidities occurring in asthma patients in Saarland and Rhineland-Palatinate. The present study offers for the first time a calculation of the chances for different comorbidities occurring dependent upon and in the context of bronchial asthma in Saarland and Rhineland-Palatinate. Asthma is often accompanied by allergic diseases like allergic rhinitis (OR 7.02) and allergic conjunctivitis (OR 4.98); non-allergic comorbidities, such as obstructive sleep apnea syndrome (OSAS) (OR 2.55) and pneumonia (OR 1.83) also show increased occurrence. Based on the findings of this study, especially allergic and respiratory comorbidities should be taken into consideration in the diagnostic and treatment strategy of bronchial asthma.

## Methods

In the present study, we analyzed the prevalence and association of clinically important comorbidities in asthmatics in Saarland and Rhineland-Palatinate. For this population-based cross-sectional study we used two data bases: (i) Association of Statutory Health Insurance Physicians Saarland (Kassenärztliche Vereinigung Saarland, KVS) and (ii) statutory health insurance fund AOK (Allgemeine Ortskrankenkasse).

### Collection of patient data to calculate prevalence rates

The pseudo-anonymized data of all patients (653,955 patient cases) who sought medical advice in the time period from 2009 to the second quarter of 2012 in the German region Saarland were provided by the KVS, which collects all diagnoses of residents who are insured with statutory insurance funds.

The provided data, which consisted of the characteristics gender, age group, and all ICD-10-coded diagnoses, were approved by the Ethics Commission of the Medical Council Saarland (Dr. Ja./Gn, 20150825). According to the Ethics Committee a consent was not necessary because the data was provided pre-anonymized. The data covered approximately 95% of all patient diagnoses, which were made by the resident physicians in Saarland. Prevalences were calculated as the ratio of asthma diagnoses and the number of insured inhabitants of Saarland (2011: 860,880). Denominator data was provided by the Federal Ministry of Health (source: https://www.bundesgesundheitsministerium.de/fileadmin/dateien/Downloads/Statistiken/GKV/Mitglieder_Versicherte/KM6_2012.xls). Subgroup analyses were conducted for sex, age and the four asthma phenotypes as defined by ICD-10 (J45.0, J45.1, J45.8 and J45.9). For convenience, we assigned all relevant comorbidities to six disease groups. Those groups were (i) allergic (ii) respiratory (iii) cardiovascular (iv) metabolic (v) mental, and vi) other diseases.

Allergic diseases represented the most important group of comorbidities of bronchial asthma. Allergic rhinitis consisted of allergic rhinopathy due to pollen, such as hay fever, and other seasonal and all-season rhinopathies. Atopic dermatitis consisted of Prurigo Besnier (L20.0), other forms of atopic eczema (L20.8), and atopic dermatitis, not otherwise specified (L20.9). Sinusitis was composed of both the acute (J01) and the chronic (J32) manifestation. Acute and chronic sinusitis and rhinopharyngitis, allergic bronchopulmonary aspergillosis, bronchiectasis, pneumonia, pulmonary embolism, and OSAS were assigned to diseases of the respiratory system. The comorbidities we defined as cardiovascular diseases were myocardial infarction, ischemic heart disease, primary and secondary hypertonia, cardiac arrhythmias, and cardiac insufficiency.

The next group consisted of comorbidities that were related to metabolic diseases. These were both types of diabetes mellitus, hyperlipoproteinaemia and other lipidaemias and adiposity.

Bronchial asthma can occur with mental illnesses. These could be social phobias, affective disorders and unspecified anxiety disorders.

In the course of bronchial asthma, patients may also suffer from cerebrovascular diseases or other disorders, like GERD, osteoporosis, and VCD.

### Collection of patient data to calculate odds ratios

The data provided by the AOK consisted of a sample of 1,155,350 patients who were insured with AOK and sought medical advice in 2012. The data consisted of three groups. Patients, who contacted the physician due to (i) asthma, (ii) COPD, and (iii) other reasons. The entire data set was not a random sample but the individuals of each group were assigned randomly. The size of each group and the proportion of cases and controls, therefore, was artificial (patients with asthma: 79,299 (38.5%); patients with COPD: 83,845 (40.7%); patients without asthma or COPD: 142,616 (69.2%); total amount of patients: 286,024). We used asthmatics as cases and patients of the third group (non-asthmatics-non-COPD patients) as controls to calculate odds ratios. The results have to be interpreted in the light of those circumstances. The data for each single patient included an ID, an age group, and diagnoses, which were encoded according to ICD-10 guidelines. Each distinct age group covered 10 years of life. After an overview, we analyzed the allergic diseases such as allergic rhinitis, atopic dermatitis, allergic conjunctivitis, drug allergy, and food allergy in the context of the four asthma subtypes according to the ICD-10 guidelines: extrinsic (J45.0), intrinsic (J45.1), mixed (J45.8), and unspecified (J45.9) asthma.

### Statistical analyses

We estimated adjusted odds ratios with 95% confidence intervals using a binary logistic regression for every comorbidity in the context of bronchial asthma. An adjustment was made for age and sex. Age was measured in 10 year classes. The data were processed using R software, version 3.1.3 (R Foundation for Statistical Computing). Statistical analyses and visualization were performed using IBM SPSS Statistics 20 (IBM, USA), GraphPad Prism 5 software (GraphPad Software, USA) and Microsoft Excel 2010 (Microsoft, USA).
